# Characterization of the l-alanine exporter AlaE of *Escherichia coli* and its potential role in protecting cells from a toxic-level accumulation of l-alanine and its derivatives

**DOI:** 10.1002/mbo3.269

**Published:** 2015-06-13

**Authors:** Seryoung Kim, Kohei Ihara, Satoshi Katsube, Hatsuhiro Hori, Tasuke Ando, Emiko Isogai, Hiroshi Yoneyama

**Affiliations:** Laboratory of Animal Microbiology, Department of Microbial Biotechnology, Graduate School of Agricultural Science, Tohoku University1-1, Amamiya-machi, Tsutsumidori, Aoba-ku, Sendai, 981-8555, Japan

**Keywords:** *Escherichia coli*, exporter, l-alanine

## Abstract

We previously reported that the *alaE* gene of *Escherichia coli* encodes the l-alanine exporter AlaE. The objective of this study was to elucidate the mechanism of the AlaE exporter. The minimum inhibitory concentration of l-alanine and l-alanyl-l-alanine in *alaE*-deficient l-alanine-nonmetabolizing cells MLA301Δ*alaE* was 4- and >4000-fold lower, respectively, than in the *alaE*-positive parent cells MLA301, suggesting that AlaE functions as an efflux pump to avoid a toxic-level accumulation of intracellular l-alanine and its derivatives. Furthermore, the growth of the *alaE*-deficient mutant derived from the l-alanine-metabolizing strain was strongly inhibited in the presence of a physiological level of l-alanyl-l-alanine. Intact MLA301Δ*alaE* and MLA301*ΔalaE*/pAlaE cells producing plasmid-borne AlaE, accumulated approximately 200% and 50%, respectively, of the [^3^H]l-alanine detected in MLA301 cells, suggesting that AlaE exports l-alanine. When 200 mmol/L l-alanine-loaded inverted membrane vesicles prepared from MLA301*ΔalaE*/pAlaE were placed in a solution containing 200 mmol/L or 0.34 *μ*mol/L l-alanine, energy-dependent [^3^H]l-alanine accumulation occurred under either condition. This energy-dependent uphill accumulation of [^3^H]l-alanine was strongly inhibited in the presence of carbonyl cyanide *m*-chlorophenylhydrazone but not by dicyclohexylcarbodiimide, suggesting that the AlaE-mediated l-alanine extrusion was driven by proton motive force. Based on these results, physiological roles of the l-alanine exporter are discussed.

## Introduction

The cytoplasmic membrane of bacteria plays a crucial role in maintaining intracellular homeostasis (Kadner 1996), that is the balance between transporter-mediated solute uptake and the efflux of their substrates (Kadner 1996). In terms of amino acid metabolism, extensive biochemical and genetic studies have identified and characterized a large number of amino acid importers (Piperno and Oxender 1968; Kay 1971; Oxender 1972; Cosloy 1973; Robbins and Oxender 1973; Guardiola et al. 1974a; Kobayashi et al. 1974); in contrast, due in part to technical difficulties, studies on amino acid exporters have lagged behind. Since the discovery of the lysine exporter, LysE, of *Corynebacterium glutamicum* (Vrljic et al. 1996), more than a dozen amino acid exporters and their homologues have been identified including, threonine, isoleucine, and glutamic acid exporters in *C. glutamicum* (Simic et al. 2001; Kennerknecht et al. 2002; Nakamura et al. 2007), homoserine, cysteine, threonine, arginine, leucine, and aromatic amino acids exporters in *Escherichia coli* (Zakataeva et al. 1999; Dassler et al. 2000; Pittman et al. 2002; Franke et al. 2003; Livshits et al. 2003; Nandineni and Gowrishankar 2004; Kutukova et al. 2005; Yamada et al. 2006; Doroshenko et al. 2007; Park et al. 2007), and the aspartate alanine exchanger in *Tetragenococcus halophilus* (Abe et al. 2002). The physiological roles of the amino acid exporters was assumed to be homeostatic maintenance of the intracellular amino acid pool, by acting as a safety valve to avoid the toxic-level accumulation of intracellular amino acids, and to export intercellular signaling molecules, such as the quorum-sensing mediator homoserine lactone (Krämer 1994; Fuqua et al. 1996; Paulsen et al. 1996; Zakataeva et al. 1999). However, experimental evidence to support these hypotheses is lacking.

Recently, we have identified the *alaE* (formerly *ygaW*) gene encoding the major l-alanine exporter in *E. coli* (Hori et al. 2011b). Interestingly, the AlaE protein contains only 149 amino acid residues, which is the smallest among the amino acid exporters identified to date, and *alaE* homologues were found to be conserved only in gamma- and alpha-proteobacteria (Hori et al. 2011b). The *alaE*-deficient mutant was found to be hypersusceptible to the l-alanine dipeptide, l-Ala-l-Ala, suggesting that the mutant accumulated a toxic level of l-alanine or alanine dipeptide (Hori et al. 2011a). Indeed, the AlaE-deficient mutant derived from an l-alanine nonmetabolizing strain accumulated over 160 mmol/L l-alanine in the presence of 6 mmol/L l-Ala-l-Ala, whereas the AlaE-overexpressing cell accumulated less than 40 mmol/L l-alanine under the same conditions (Hori et al. 2011b). However, it is not clear how the *alaE* defect renders the cells hypersusceptible to the l-alanine dipeptide. Certain dipeptides including l-leucyl-glycine, l-leucyl-l-leucine, glycyl-l-leucine, l-leucyl-l-tyrosine, and l-prolyl-l-leucine were reported to inhibit the growth of an l-leucine auxotroph (Simmonds et al. 1951), and l-alanyl-l-glutamine and glycyl-l-tyrosine were shown to inhibit the growth of peptidase-deficient *E. coli* cells (Hayashi et al. 2010). These results suggest that a threshold accumulation of certain dipeptides might be toxic to the cells and, therefore, the amino acid exporters might play a role in the removal of these compounds and/or their derivatives, thereby protecting the cells.

In order to elucidate the physiological roles of the amino acid exporters, it is essential to gain a thorough understanding of their function. For this purpose, we used the l-alanine exporter AlaE of *E. coli* as a model, and analyzed the impact of AlaE deficiency on the viability of the cells and the transport properties using inverted membrane vesicles. The results suggested that the l-alanine exporter acts as a “safety valve (or drain valve)” in the maintenance of an intracellular l-alanine pool and prevents the toxic-level accumulation of l-alanine and its analogues.

## Experimental Procedures

### Bacterial strains, plasmids, and growth conditions

The *E. coli* strains and plasmids used in this study are listed in Table1. Cells were grown aerobically at 37°C in l-broth containing 1% tryptone (w/v), 0.5% yeast extracts (w/v), and 0.5% NaCl (w/v) (pH 7.2) or minimal medium (Fisher et al. 1981) containing 22 mmol/L glucose, 7.5 mmol/L (NH_4_)_2_SO_4_, 1.7 mmol/L MgSO_4_, 7 mmol/L K_2_SO_4_, 22 mmol/L NaCl and 100 mmol/L sodium phosphate (pH 7.1). d-alanine (50 *μ*g mL^−1^), l-alanine (50 *μ*g mL^−1^), gentamicin (6.25 *μ*g mL^−1^), kanamycin (6.25 *μ*g mL^−1^), and chloramphenicol (12.5 *μ*g mL^−1^) were supplemented as needed. Growth was monitored by measuring absorbance at 660 nm (*A*_660_).

**Table 1:** List of strains and plasmids used

Strains or plasmids	Relevant properties	Source or reference
*Escherichia coli*		
JM109	*recA*1, *endA*1, *gyrA*96, *thi*, *hsdR*17(r_k_^−^ m_k_^+^), *e*14^−^(*mcrA*^−^), *supE*44, *relA*1, Δ(*lac-proAB*)/F’[*traD*36, *proAB*^+^, *lacI*^q^, *lac*ZΔM15]	Laboratory strain
MLA301	MG1655 *alr*::*FRT*, *dadX*::*FRT*, *yfdZ*::*FRT*, *avtA*::GM, yfbQ::KM	Hori et al. (2001a)
MLA301Δ*alaE*	MLA301 derivative with a deletion in the *alaE* gene	Hori et al. (2001b)
MG1655	wild-type	Hori et al. (2001b)
MG1655Δ*alaE*	MG1655 derivative with a deletion in the *alaE* gene	Hori et al. (2001b)
*Plasmid*		
pSTV29	CP^r^, *lacZ*, p15A ori	Takara
pAlaE	pSTV29 harboring a 1.0-kb PCR fragment of the *alaE* gene	Hori *et al*., (2011b)

GM, gentamicin; KM, kanamycin; CP, chloramphenicol; AP, ampicillin.

### Determination of MIC of amino acids and l-Ala-l-Ala

MICs of amino acids and l-alanine analogues in MLA301 and MLA301Δ*alaE* were determined using the agar dilution method on minimal medium. The effect of l-Ala-l-Ala and l-alanine on the growth of MG1655 and its *alaE*-deficient mutant MG1655Δ*alaE* was assessed as follows: a 10-fold serially diluted fully grown cell suspension (5 *μ*L) was spotted on minimal agar medium impregnated with 0.5–4.0 mmol/L l-Ala-l-Ala or 2.5–20 mmol/L l-Ala and incubated at 37°C for 40 h.

### l-alanine accumulation in intact cells

Cells were grown in l-broth containing d-alanine (50 *μ*g mL^−1^) and appropriate antibiotics as described above at 37°C overnight, and harvested by centrifugation at 8900*g* for 10 min. Pellets were washed twice with minimal medium containing d-alanine (50 *μ*g mL^−1^), gentamicin (6.25 *μ*g mL^−1^), and kanamycin (6.25 *μ*g mL^−1^) and suspended in the same medium adjusting *A*_660_ to 0.5. After incubation at 37°C for 2 h, cells were collected by centrifugation as described above, suspended in the same ice-cold minimal medium adjusting the cell density to *A*_660_ of 1.0, and kept on ice until use. A plastic tube containing 1.5 mL of cell suspension in the presence of chloramphenicol (300 *μ*g mL^−1^) was preincubated at 37°C for 10 min. The reaction was initiated by adding a 1.5 mL aliquot of the same minimal medium containing 20 *μ*mol/L l-[2,3-^3^H]alanine (60 Ci mmol^−1^, American Radiolabeled Chemical, Saint Louis, MO, USA), and terminated by filtering 100 *μ*L aliquots through a membrane filter with a pore size of 0.22 *μ*m followed by washing twice with 3 mL each of prewarmed medium. The membrane filter was placed in a plastic vial and immersed in 6 mL of the scintillant Filter-Count (PerkinElmer, Waltham, MA, USA). The radioactivity was counted after 24 h by a liquid scintillation counter, SLC-5001 (Hitachi Aloka Medical, Mitaka, Japan).

### Preparation of inverted membrane vesicles

Inverted membrane vesicles were prepared as described previously (Altendorf and Staehelin 1974). Briefly, cells grown in 150 mL of l-broth at 37°C overnight were diluted with 1.35 L of fresh medium and the mixture was incubated on a Bio-shaker BR-300LF (TAITEC Co., Koshigaya, Japan) shaking at 100 reciprocals per minute until the *A*_660_ reached 1.0. Cells were collected by centrifugation at 8900*g* for 10 min at 4°C and washed twice with a solution containing 50 mmol/L potassium phosphate and 5 mmol/L MgSO_4_ (pH 7.0). The cells were suspended in a solution containing 50 mmol/L potassium phosphate, 5 mmol/L MgCl_2_, 1 mmol/L dithiothreitol (DTT), and 20% glycerol (v/v) (pH 7.0) and passed through a French pressure cell (AVESTIN, Ottawa, Canada) at 82.8 MPa. Unbroken cells and large cell debris were removed by centrifugation at 39,000*g* for 20 min at 4°C. Next, the supernatant fraction was centrifuged (Beckman XL-90, Brea, CA, USA) at 231,000*g* for 1.5 h at 4°C to collect the inverted membrane vesicles. Pellets were resuspended in the same solution adjusting the protein concentration to 25 mg mL^−1^, divided into small aliquots, quickly frozen with liquid nitrogen_,_ and then kept at −80°C until use. For the preparation of l-alanine-loaded inverted vesicles, cells were washed twice with a solution containing 50 mmol/L potassium phosphate, 5 mmol/L MgSO_4_, and 200 mmol/L l-alanine (pH 7.0) and then suspended in a solution containing 50 mmol/L potassium phosphate, 5 mmol/L MgCl_2_, 1 mmol/L DTT, 20% glycerol (v/v), and 200 mmol/L l-alanine (pH 7.0). The suspension was subjected to the French pressure cell treatment as described above.

### Uptake assay with inverted membrane vesicles

Uptake of [^3^H]l-alanine into inverted membrane vesicles was carried out as follows. After incubation of 20 *μ*L (0.5 mg protein equivalent) of the membrane vesicles for 2 min in a 25°C water bath, the reaction was initiated by adding 80 *μ*L of a solution containing [^3^H]l-alanine with or without 3.15 mmol/L NADH in 50 mmol/L potassium phosphate and 5 mmol/L MgSO_4_ (pH 7.0). An aliquot of the mixture was filtered through a 0.22 *μ*m-pore size membrane filter (Millipore Co., Billerica, MA, USA) at the indicated time intervals, and washed twice with 3 mL of the same buffer. Next, the membrane filters were immersed in 6 mL of the scintillant Filter-Count (PerkinElmer) and the radioactivity was counted after 24 h using a liquid scintillation counter SLC-5001 (Hitach Aloka Medical, Mitaka, Japan).

### Effect of CCCP and DCCD on l-alanine accumulation in inverted membrane vesicles

The 200 mmol/L l-alanine-loaded inverted vesicles (0.5 mg protein equivalent in 20 *μ*L) were preincubated at 25°C for 100 sec, then 2 *μ*L of 125 mmol/L NADH was added and the vesicles were incubated for an additional 20 sec at 25°C. The reaction was initiated by adding a solution containing 50 mmol/L potassium phosphate (pH 7.0), 5 mmol/L MgSO_4_ and carbonyl cyanide *m*-chlorophenylhydrazone (CCCP, final 20 *μ*mol/L) or dicyclohexylcarbodiimide (DCCD, final 50 *μ*mol/L), and [^3^H]l-alanine (38 Ci mmol^−1^, Moravek Biochemical, Brea, CA, USA; final, 0.54 *μ*mol/L). After incubation for 5 min at 25°C, an aliquot of the reaction mixture was filtered and washed as described above with prewarmed buffer (50 mmol/L potassium phosphate (pH 7.0) and 5 mmol/L MgSO_4_). Radioactivity was measured as described above. A net AlaE-dependent [^3^H]l-alanine accumulation in the vesicles was obtained by subtracting the accumulation in the vesicles prepared from *alaE*-deficient cells from that obtained using *alaE*-overexpressing cells.

### Effect of selected amino acids and l-alanine analogues on the AlaE-mediated l-alanine accumulation in inverted membrane vesicles

The 200 mmol/L l-alanine-loaded inverted vesicles (0.5 mg protein equivalent in 20 *μ*L) were preincubated at 25°C for 100 sec, then 2 *μ*L of 125 mmol/L NADH was added and the vesicles were incubated for an additional 20 sec at 25°C. The reaction was initiated by adding 78 *μ*L of a solution containing [^3^H]l-alanine (final 0.54 *μ*mol/L) supplemented with the selected amino acid in 50 mmol/L potassium phosphate and 5 mmol/L MgSO_4_ (pH 7.0). The concentration of d-alanine ranged from 100 *μ*mol/L to 2.5 mmol/L. Amino acids to be tested were added to a final concentration of 100 *μ*mol/L. The pH of stock amino acid solutions was adjusted to 7.0, except for tyrosine, which was adjusted to pH 7.2. After 5 min of incubation, an aliquot of the mixture was filtered and washed as described above with prewarmed buffer, and the radioactivity was counted as described above. AlaE-dependent [^3^H]l-alanine accumulation in the vesicles was obtained as described in the previous section and the value in the absence of a competitor was set to 100%.

### Protein quantification

Protein concentrations were determined by the method of Lowry et al. (1951).

## Results

### l-alanine accumulation in intact cells producing different levels of AlaE

**Figure 1:**
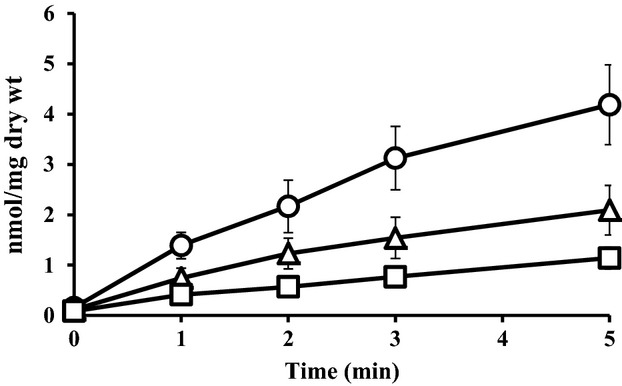
Accumulation of [^3^H]l-alanine in intact cells. Cells were grown in minimal medium and suspended in the same medium. The transport assay was initiated by adding [^3^H]l-alanine (60 Ci mmol^−1^) to a final concentration of 10 *μ*mol/L and then incubated at 37°C. An aliquot (100 *μ*L) of the cell suspension was filtered through a membrane filter with a 0.22 *μ*m-pore size, and then washed twice with 3 mL each of pre-warmed minimal medium. Values presented are the means of three independent experiments with standard deviations. Symbols: ▵, MLA301; ○, MLA301Δ*alaE*; □, MLA301Δ*alaE*/pAlaE.

As the *alaE* gene was identified to encode the l-alanine exporter (Hori et al. 2011b), we first determined time course accumulation of [^3^H]l-alanine in intact cells either producing wild-type AlaE (MLA301), lacking the *alaE* gene (MLA301Δ*alaE*), or MLA301Δ*alaE*/pAlaE producing plasmid-borne AlaE (Fig.1). The MLA301Δ*alaE* cells accumulated l-alanine in a time-dependent manner reaching 4.19 nmol (mg dry weight)^−1^ at 5 min. This may have been attributable to the functioning of active l-alanine importers and a nonfunctioning exporter. The MLA301 cells also accumulated l-alanine linearly reaching 2.09 nmol (mg dry weight)^−1^ at 5 min, which was approximately one-half that of the MLA301Δ*alaE* cells. The *alaE*-deficient mutant harboring pAlaE accumulated about 55% of the amount of l-alanine observed in MLA301 cells after 5 min, and that was approximately equivalent to one-quarter of that in the AlaE-deficient cells. Although the level of intracellular l-alanine is determined as the balance of the uptake and efflux of l-alanine, the different levels of l-alanine accumulation in these strains was interpreted to be as a result of the presence or absence of the *alaE* gene. These results are consistent with earlier observations (Hori et al. 2011b).

### Susceptibility of alaE-deficient mutants to amino acids and l-alanyl- l-alanine (l-Ala-l-Ala)

The above results suggested that AlaE exports l-alanine out of the cells, and raised the question of what could be a physiological role of the l-alanine exporter. A plausible interpretation could be that the exporter protects the cells from a toxic-level accumulation of l-alanine and possibly its derivatives. To test this possibility, we have determined the MIC of various amino acids and the l-Ala-l-Ala dipeptide (Table2). Among the amino acids tested, the MIC of l-alanine in the *alaE*-deficient mutant was 1.25 mg mL^−1^, whereas the MIC in the AlaE-producing wild-type cells was 5 mg mL^−1^, suggesting that the AlaE exporter protected the cells from a toxic-level accumulation of l-alanine. Interestingly, *alaE* deficiency exerted a striking impact on the susceptibility of the cells to the dipeptide l-Ala-l-Ala, with MICs of 2.5 *μ*g mL^−1^ and >10 mg mL^−1^ in MLA301Δ*alaE* and MLA301, respectively, over a 4000-fold difference. The MICs of most of the other amino acids, including *β*-alanine, in the *alaE*-deficient mutant appeared comparable with those in the *alaE*-positive cells. Notably, the MIC of d-alanine appeared identical, 20 mg mL^−1^, in both strains, suggesting that AlaE did not export d-alanine. Alternatively, it is possible that d-alanine accumulation is not harmful to the *E. coli* cells.

**Table 2:** MICs of amino acids and l-alanyl-l-alanine in MLA301 and MLA301Δ*alaE*

Strains	MIC (mg mL^−1^)											
	l-Ala	l-Cys	l-His	l-Ile	l-Leu	l-Met	l-Phe	l-Ser	l-Trp	l-Asn	l-Arg	l-Tyr
MLA301	5	0.5	10	10	2.5	40	30	10	15	30	20	>0.4
MLA301Δ*alaE*	1.25	0.5	10	10	5	40	30	10	15	30	20	>0.4
	l-Asp	l-Gln	l-Glu	l-Lys	l-Pro	l-Thr	Gly	l-Val	l-Ala- l-Ala	d-Ala	*β*-Ala	d-Ser
MLA301	20	40	20	80	120	10	5	0.313	>10	20	60	10
MLA301Δ*alaE*	20	40	20	80	120	10	5	0.156	0.0025	20	60	10

**Figure 2:**
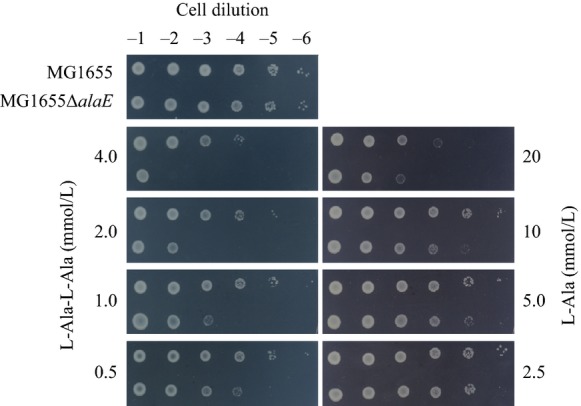
Effect of l-alanine and l-Ala-L-Ala on the growth of the wild-type strain and its *alaE*-deficient derivative. A fully grown cell suspension was serially diluted 10-fold and 5-*μ*L aliquots were spotted onto minimal medium supplemented with 0.5–4.0 mmol/L l-Ala-l-Ala (left panel) or 2.5–20 mmol/L l-Ala (right panel). The top-left panel represents control medium without supplement. Plates were incubated at 37°C for 40 h. Strains used: MG1655, *alaE*-positive parent strain; MG1655Δ*alaE*, *alaE*-deficient isogenic derivative.

In addition, we assessed the effect of l-Ala-l-Ala and l-alanine on the growth of the *alaE*-deficient cells derived from an l-alanine-metabolizing wild-type strain by plating a 10-fold serially diluted cell suspension, and the results were compared with that of the *alaE*-positive parent cells (Fig.2). Both the *alaE*-deficient cells and the parent cells grew up to the 10^−6^ dilution in the control plate free from l-Ala-l-Ala and l-alanine. When the plate was impregnated with 4.0 mmol/L l-Ala-l-Ala, the parent cells grew up to a dilution of 10^−4^, a difference of two orders of magnitude from that on the l-Ala-l-Ala-free medium. In contrast, the *alaE*-deficient cells grew only at the 10^−1^ dilution, a difference of five orders of magnitude from the control plate. As the l-Ala-l-Ala concentration was decreased to 2.0, 1.0, and 0.5 mmol/L, the effect of l-Ala-l-Ala was reduced, as would be expected, however, the difference in growth when compared with the control plate was noticeable even at 0.5 mmol/L. Similar experiments were carried out in plates impregnated with l-alanine at concentrations of 2.5, 5.0, 10, and 20 mmol/L. The *alaE*-deficient cells showed higher susceptibility to l-alanine than the parent cells, yet the growth inhibitory effect was less significant when compared with that of l-Ala-l-Ala, suggesting that the primary substrate of the AlaE exporter may be alanine derivatives including l-Ala-l-Ala, but we cannot rule out a possibility that the primary substrate of the AlaE may be l-alanine.

### l-alanine accumulation in inverted membrane vesicles under a downhill solute gradient

**Figure 3:**
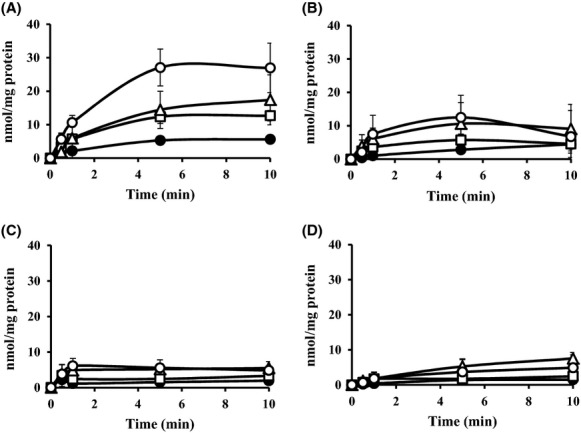
Accumulation of [^3^H]l-alanine (60 Ci mmol^−1^) in the inverted membrane vesicles under a downhill solute potential. The inverted membrane vesicles were prepared from MLA301Δ*alaE*/pAlaE (A and B) and MLA301Δ*alaE* (C and D). [^3^H]l-alanine accumulation was assayed in the presence (A and C) and absence (B and D) of 2.5 mmol/L NADH. Extravesicular l-alanine concentrations for the transport assays are 50 mmol/L (●), 100 mmol/L (□), 150 mmol/L (▵) and 200 mmol/L (○). Values presented are the means of more than three independent experiments with standard deviations.

To demonstrate l-alanine export directly in the cell-free system, we determined [^3^H]l-alanine accumulation in inverted membrane vesicles prepared from MLA301Δ*alaE* and MLA301Δ*alaE*/pAlaE cells under a downhill solute gradient. Experiments were carried out to examine four parameters: (i) time course accumulation of l-alanine, (ii) the impact of the presence or absence of the AlaE exporter, (iii) the effect of the energy source that drives the active efflux of l-alanine, and (iv) the effect of the extravesicular l-alanine concentration. The time course of l-alanine accumulation was first measured in vesicles prepared from cells lacking AlaE in the presence of an energy source and 200 mmol/L extravesicular l-alanine. As shown in Figure3C, the vesicles accumulated only a basal level of l-alanine. In contrast, similar experiments using membrane vesicles prepared from cells with intact AlaE accumulated 27.1 nmol l-alanine (mg protein)^−1^ in 5 min, and the level was maintained for at least 10 min in the presence of 200 mmol/L extravesicular l-alanine (Fig.3A). This intravesicular accumulation of l-alanine appears to have been mediated by AlaE, as the vesicles prepared from MLA301Δ*alaE* accumulated a negligible amount of l-alanine under the same conditions (Fig.3C). When the energy source used to drive the active efflux was depleted, the vesicles prepared from cells producing intact AlaE accumulated l-alanine to a level of 12.5 nmol (mg protein)^−1^ in 5 min (Fig.3B), which was less than a half of the amount accumulated in the energized vesicles at 200 mmol/L extravesicular l-alanine (Fig.3A). This level of l-alanine accumulation may have been the result of downhill facilitated diffusion of the solute through the l-alanine carriers even without an energy source. Thus, it is clear that the vesicles prepared from cells with intact AlaE accumulated l-alanine in an energy-dependent manner. As the substrate concentration was varied from 50 to 200 mmol/L, the AlaE-positive vesicles prepared from AlaE-producing cells accumulated an increasing amount of l-alanine in response to the extravesicular l-alanine concentration as expected (Fig.3A). Attempts have been made to calculate a transport *K*_m_ of l-alanine; however, it was difficult to get a stable value mainly due to slightly different properties of the inverted membrane vesicles from a batch to another.

### l-alanine accumulation in inverted membrane vesicles under an equilibrium solute state

**Figure 4:**
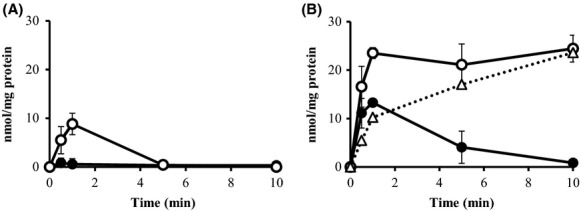
Accumulation of [^3^H]l-alanine (60 Ci mmol^−1^) in the l-alanine-loaded inverted membrane vesicles under an equilibrium solute potential. l-alanine-loaded inverted membrane vesicles were prepared from MLA301Δ*alaE*/pAlaE and MLA301Δ*alaE*. The extravesicular l-alanine concentration for the transport assay was 200 mmol/L, which was equivalent to that of intravesicular l-alanine (200 mmol/L). Transport assays were performed without NADH (A) and with 2.5 mmol/L NADH (B). Dotted line in (B) shows AlaE-mediated l-alanine accumulation obtained by subtracting the values in MLA301Δ*alaE* from those in MLA301Δ*alaE*/pAlaE in the presence of NADH. Values presented are means of three independent experiments with standard deviations. Symbols: ○, MLA301Δ*alaE*/pAlaE; ●, MLA301Δ*alaE*.

As the above-described experiments were carried out under conditions where the AlaE exporter catalyzed a downhill movement of l-alanine according to the substrate chemical potential, we next examined whether AlaE catalyzes l-alanine movement under conditions in which the internal and external solute concentrations were in equilibrium. For this purpose, we prepared the inverted membrane vesicles in the presence of 200 mmol/L nonradioactive l-alanine. We then determined the [^3^H]l-alanine accumulation in the loaded vesicles in the presence of 200 mmol/L extravesicular radiolabeled l-alanine. The l-alanine-loaded vesicles prepared from MLA301Δ*alaE*/pAlaE showed a clear energy-dependent accumulation of [^3^H]l-alanine, reaching a plateau at 23.5 nmol (mg protein)^−1^ in 1 min (Fig.4B). In contrast, the vesicles prepared from MLA301Δ*alaE* accumulated only 13.3 nmol l-alanine (mg protein)^−1^ in 1 min followed by a slow leakage in the presence of NADH (Fig.4B). As this transient l-alanine accumulation likely occurred in an AlaE-independent manner, these values were subtracted from that of AlaE-mediated l-alanine accumulation. The values shown by a dotted line in Figure4B were likely attributable to actual AlaE-mediated transport activity. It is of interest to note that a transient increase in intravesicular l-alanine was similarly observed in the absence of NADH (Fig.4A).

### l-alanine accumulation in inverted membrane vesicles under an uphill solute gradient

**Figure 5:**
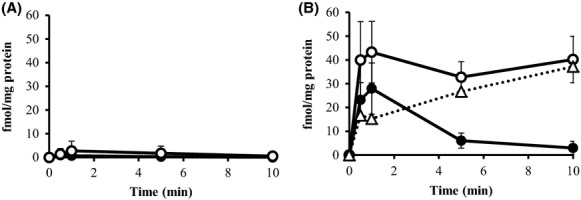
Accumulation of [^3^H]l-alanine (60 Ci mmol^−1^) in the l-alanine-loaded inverted membrane vesicles under an uphill solute potential. Inverted membrane vesicles were prepared from MLA301Δ*alaE*/pAlaE and MLA301Δ*alaE*. The extravesicular l-alanine concentration for the transport assay was 0.34 *μ*mol/L. Transport assays were performed without NADH (A) and with 2.5 mmol/L NADH (B). Dotted line in (B) shows AlaE-mediated l-alanine accumulation obtained by subtracting the values in MLA301Δ*alaE* from those in MLA301Δ*alaE*/pAlaE in the presence of NADH. Values presented are the means of three independent experiments with standard deviations. Symbols: ○, MLA301Δ*alaE*/pAlaE; ●, MLA301Δ*alaE*.

We next determined the [^3^H]l-alanine accumulation in the 200 mmol/L l-alanine-loaded membrane vesicles in the presence of only 0.34 *μ*mol/L extravesicular [^3^H]l-alanine, a setup for an outward l-alanine chemical potential. The membrane vesicles prepared from MLA301Δ*alaE*/pAlaE rapidly accumulated [^3^H]l-alanine, reaching 43.3 fmol (mg protein)^−1^ within 1 min in the presence of NADH, and the level of labeled l-alanine was steadily maintained for at least 10 min (Fig.5B). In contrast, only a trace amount of l-alanine accumulation was observed in the absence of the energy source (Fig.5A). The vesicles prepared from MLA301Δ*alaE* showed a transient increase in intravesicular l-alanine, reaching 28 fmol (mg protein)^−1^ in 1 min in the presence of NADH, and then declined to the basal level after 10 min (Fig.5B). This energy-dependent l-alanine accumulation is likely due to exporters other than AlaE as reported earlier (Hori et al. 2011b). Subtraction of the l-alanine accumulation in MLA301Δ*alaE* vesicles from that of MLA301Δ*alaE*/pAlaE showed the actual AlaE-catalyzed energy-dependent uphill movement of l-alanine (Fig.5B, dotted line). Taken together, these results unequivocally demonstrate that AlaE catalyzes the active export of l-alanine in an energy-dependent manner.

### Effect of CCCP and DCCD on the active transport of l-alanine by inverted membrane vesicles

**Figure 6:**
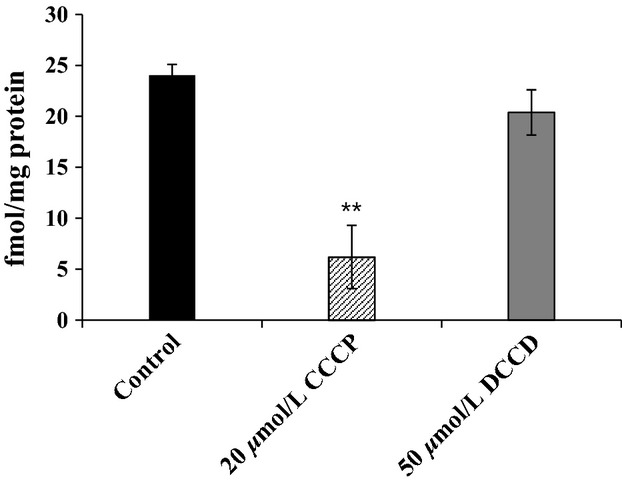
Effect of CCCP and DCCD on the accumulation of [^3^H]l-alanine in the l-alanine-loaded inverted membrane vesicles under an uphill solute potential. [^3^H]l-alanine accumulation was determined after 5 min of incubation as described in Materials and Methods. The AlaE-mediated [^3^H]l-alanine (38 Ci mmol^−1^) accumulation was calculated by subtracting values obtained with MLA301Δ*alaE* vesicles from those in MLA301Δ*alaE*/pAlaE in the presence of NADH. Solid black bar, control; hatched bar, 20 *μ*mol/L CCCP; solid gray bar, 50 *μ*mol/L DCCD. Values presented are the means of three independent experiments with standard deviations (unpaired Student *t-*test, **<0.001).

To evaluate the nature of the energy source that drives the AlaE-mediated active export of l-alanine, we determined the [^3^H]l-alanine accumulation in the 200 mmol/L l-alanine-loaded membrane vesicles in the presence and absence of 20 *μ*mol/L of carbonyl cyanide CCCP under an uphill solute gradient. As shown in Figure6, accumulation of [^3^H]l-alanine in the l-alanine-loaded MLA301Δ*alaE*/pAlaE vesicles decreased by approximately 74% after 5 min in the presence of CCCP compared to that obtained in the absence of CCCP. In contrast, when 50 *μ*mol/L of *N*, *N*’-DCCD, an ATPase inhibitor (Hirata et al. 1974; Muller et al. 1987; Hermolin and Fillingame 1989), was present in the assay system, accumulation of [^3^H]l-alanine appeared comparable to that without inhibitor suggesting that ATP does not drive this active export system. An additional energy source conceivable may be the electrochemical potential of Na^+^ ion. However, this possibility appeared less likely as addressed in the discussion section. These results indicate that AlaE catalyzes the active export of l-alanine most likely utilizing proton electrochemical potential.

### Effect of amino acids on the AlaE-mediated accumulation of l-alanine in inverted membrane vesicles

**Figure 7:**
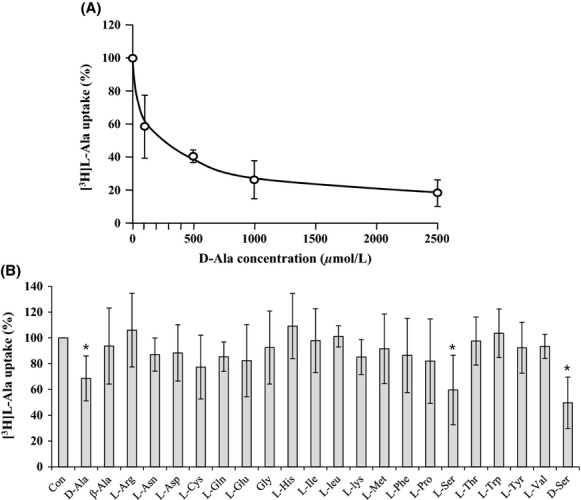
Effect of unlabeled amino acids on the AlaE-mediated active transport of l-alanine. Accumulation of [^3^H]l-alanine (38 Ci mmol^−1^) in 200 mmol/L l-alanine-loaded membrane vesicles was determined as described in Materials and Methods in the presence of various amounts of unlabeled d-alanine (A) and various amino acids (0.1 mmol/L) (B). [^3^H]l-alanine accumulation was calculated by subtracting the values obtained with MLA301Δ*alaE* vesicles from those in MLA301Δ*alaE*/pAlaE in the presence of NADH. The value obtained in the absence of unlabeled amino acid was set to 100%. The result shows an average of at least three independent experiments with standard deviations (unpaired Student *t*-test, *<0.05).

To determine the transport specificity of AlaE, we next examined whether other amino acids exert any impact on [^3^H]l-alanine accumulation in the l-alanine-loaded membrane vesicles. As a first step, we determined the effect of d-alanine, an optical isomer of l-alanine, on the [^3^H]l-alanine accumulation in the vesicles under an uphill l-alanine gradient (Fig.7A). d-alanine at a concentration of 0.1, 0.5, 1.0, and 2.5 mmol/L inhibited [^3^H]l-alanine accumulation by 41, 59, 74, and 82%, respectively, compared to the value without d-alanine. Thus, d-alanine was found to inhibit [^3^H]l-alanine accumulation in a dose-dependent manner. Based on that a 50% inhibitory concentration of d-alanine was calculated to be around 300 *μ*mol/L under the conditions tested. Accordingly, we then determined the effect of various amino acids, including the l-alanine analogues, *β*-alanine, d-serine, and l-serine at a concentration of 100 *μ*mol/L on the accumulation of l-alanine. Among the amino acids tested, l-serine and d-serine equally inhibited [^3^H]l-alanine accumulation, of which inhibition levels were slightly stronger than d-alanine. Other amino acids tested here, including *β*-alanine, showed only a marginal effect (Fig.7B).

## Discussion

In this study we characterized the function of the AlaE amino acid exporter of *E. coli* by determining (i) the biochemical analyses of AlaE-mediated l-alanine export by means of inverted membrane vesicles, and (ii) the impact of the presence and absence of AlaE on the MIC as well as the growth inhibition of l-alanine and l-Ala-l-Ala. The inverted membrane vesicles prepared from AlaE-producing cells accumulated l-alanine under downhill substrate gradients, set to l-alanine concentrations of 50–200 mmol/L, in an energy-dependent manner, whereas the vesicles prepared from the AlaE-negative cells accumulated a negligible amount (Fig.3). This result seems reasonable because the extravesicular l-alanine concentration was set to over the MIC of l-alanine. Therefore, these alanine concentrations seem vitally hazardous to the cells. Similarly, AlaE-positive vesicles accumulated l-alanine under substrate equilibrium set to 200 mmol/L l-alanine, whereas the AlaE-negative vesicles only transiently accumulated l-alanine in the presence of NADH (Fig.4B). This transient l-alanine accumulation could be attributable to other amino acid exporters such as YtfF, YddG, and YeaS (Hori et al. 2011b). The decline of intravesicular l-alanine after the initial uptake might have been due to the l-alanine importers CycA and LIV (Robbins and Oxender 1973; Guardiola et al. 1974b; Collins et al. 1976). The AlaE-positive vesicles transiently accumulated l-alanine under equilibrium in the absence of NADH (Fig.4A), but this was not observed in AlaE-negative vesicles (Fig.4A). Therefore, this transient accumulation of l-alanine (Fig.4A) was likely mediated by AlaE via the substrate exchange reaction, analogous to the lactose and melibiose permeases in the absence of an energy source (Lombardi and Kaback 1972; Kaczorowski and Kaback 1979; Garcia et al. 1983; Bassilana et al. 1987; Guan and Kaback 2006). Under the uphill substrate gradient set to 200 mmol/L intravesicular l-alanine, the AlaE-positive vesicles accumulated about 40 fmol (mg protein)^−1^ l-alanine confirming that the active transport occurred even under an uphill substrate concentration. All these results unequivocally demonstrated that AlaE acts as an alanine exporter.

Bacterial active transporters may be driven by the energy from chemicals, light, or that from a concentration gradient of specific ions or solutes (Bert and Wil 1993; Saier 2000). Amino acid exporters including YeaS (Kutukova et al. 2005), ThrE (Simic et al. 2001), and RhtA (Livshits et al. 2003), were reportedly driven by proton motive force based on experiments only using intact cells. However, the results may be equivocal. In contrast, CydDC of *E. coli* has been shown to export cysteine at the expense of ATP (Pittman et al. 2002). We demonstrated here using the inverted membrane vesicles that accumulation of [^3^H]l-alanine by AlaE was strongly inhibited by CCCP but not by DCCD, suggesting that AlaE was energized by the proton motive force (Fig.6). Still, one cannot rule out a possibility that the electrochemical potential of Na^+^ energizes AlaE. To address this issue, we determined [^3^H]l-alanine accumulation into the intact cells in a solution containing 100 mmol/L potassium phosphate and 5 mmol/L MgCl_2_ (pH 7.1). Our preliminary result showed that the MLA301Δ*alaE*/pAlaE cells accumulated a significantly low level of l-alanine compared with that in MLA301Δ*alaE* under the same conditions (data not shown). The levels were comparable to those observe with minimal medium. Addition of 1 mmol/L or 10 mmol/L NaCl to the above assay system did not show detectable difference. Thus it is highly likely that AlaE was energized by the proton motive force.

A question that remains to be answered is what could be the advantages to the cells to export an important nutrient, l-alanine, by the expenditure of cellular energy. If AlaE only functions as a drain valve to avoid a toxic-level accumulation of l-alanine, the overflow drain channel by facilitated diffusion should be sufficient for that purpose. Such a case has been reported in the l-glutamic acid exporter in *C. glutamicum* (Nakamura et al. 2007). Two plausible explanations may be conceivable to explain this issue. (i) The cells must export l-alanine forcefully by the expenditure of cellular energy when the extracellular l-alanine concentration exceeds a toxic level of intracellular l-alanine. However, it is less likely that the bacteria face such high l-alanine environments. In fact, the MIC of l-alanine in the AlaE-deficient cells was 1.25 mg mL^−1^ or 1.4 × 10^−2^ mol/L, which was only fourfold lower than that of the AlaE-positive parent cells, 5 mg mL^−1^ or 5.6 × 10^−2^ mol/L. Instead, it is possible that AlaE exports a toxic level of intracellularly generated l-alanine by degrading l-alanine-containing peptides, and in fact, intracellular l-alanine was found to reach over 100 mmol/L in the presence of 6 mmol/L l-Ala-l-Ala (Hori et al. 2011b). (ii) Alternatively, the primary role of AlaE could be to extrude toxic l-alanine derivatives including l-Ala-l-Ala. In fact, the MIC of l-Ala-l-Ala in the AlaE-deficient cells was 0.0025 mg mL^−1^ or 1.56 × 10^−5^ mol/L, over 4000-fold lower than that in the AlaE-positive parent cells. As 1.56 × 10^−5^ mol/L of l-Ala-l-Ala appeared toxic, it is advantageous to the cells to extrude l-Ala-l-Ala or metabolites derived from l-Ala-l-Ala at the expense of cellular energy. Excess accumulation of l-alanine and/or its derivatives above a threshold level could cause metabolic imbalance and growth inhibition.

It was reported that certain dipeptides, including l-leucyl-glycine, l-leucyl-l-leucine, glycyl-l-leucine, l-leucyl-l-tyrosine, and l-prolyl-l-leucine, inhibited the growth of an l-leucine auxotroph (Simmonds et al. 1951), and l-alanyl-l-glutamine and glycyl-l-tyrosine inhibited the growth of peptidase-deficient *E. coli* cells (Hayashi et al. 2010). Therefore, accumulation of these compounds over a threshold level may be toxic to the cells. The observation that the expression of *alaE* is inducible in the presence of a low concentration, 1 mmol/L, of l-Ala-l-Ala strongly supports the above notion that the l-alanine efflux transporter, AlaE, plays a crucial role as a life saving safety valve in the *E. coli* cells. If this scenario was valid, l-Ala-l-Ala would be expected to cause growth inhibition of *alaE*-deficient l-alanine metabolizing cells. In fact, growth of the AlaE-negative cells was inhibited in the presence of physiological concentrations of l-Ala-l-Ala, 0.5–4 mmol/L (Fig.2). Recently, multidrug exporters have been reported to possess dipeptides export activity (Hayashi et al. 2010). Furthermore, several enterobacteria were found to produce cyclic dipeptides, cyclo(ΔAla-l-Val) and cyclo(l-Pro-l-Tyr), in their culture supernatants, which may have a role in cell-to-cell communication (Holden et al. 1999). Hence, it is tempting to speculate that AlaE may have a function to export these signaling mediators.

Taken together, the results presented in this study unequivocally demonstrated that AlaE catalyzes the active export of l-alanine and possibly its derivatives, including l-alanine dipeptide, and functions as a “safety valve” to protect the cells from a toxic-level accumulation of l-alanine and its derivatives.
